# Taxonomic revision of the endemic Cameroonian freshwater crab genus *Louisea* Cumberlidge, 1994 (Crustacea, Decapoda, Brachyura, Potamonautidae), with descriptions of two new species from Nkongsamba and Yabassi

**DOI:** 10.3897/zookeys.881.36744

**Published:** 2019-10-17

**Authors:** Pierre A. Mvogo Ndongo, Thomas von Rintelen, Neil Cumberlidge

**Affiliations:** 1 Department of Management of Aquatic Ecosystems, Institute of Fisheries and Aquatic Sciences, University of Douala at Yabassi, PO. BOX. 7236 Douala-Bassa, Cameroon Leibniz-Institut für Evolutions und Biodiversitätsforschung Berlin Germany; 2 Museum für Naturkunde, Leibniz-Institut für Evolutions und Biodiversitätsforschung, Invalidenstrasse 43, 10115 Berlin, Germany University of Douala at Yabassi Douala-Bassa Cameroon; 3 Department of Biology, Northern Michigan University, Marquette, MI, 49855-5376, USA Northern Michigan University Marquette United States of America

**Keywords:** Cameroon, Crustacea, identification key, new species, phylogeny, Potamoidea, redescription, taxonomic revision

## Abstract

The taxonomy of the freshwater crab genus *Louisea* Cumberlidge, 1994, is reviewed based on type material and newly obtained specimens from three different localities in southwestern Cameroon. The genus is endemic to Cameroon and previously included two species: *L.edeaensis* (Bott, 1969) (type species) from Lake Ossa wetland complex (altitudes below 400 m asl) and *L.balssi* (Bott, 1959) from Kumba and Mt. Manengouba (altitudes above 1300 m asl). Here two new species of *Louisea* are described based on morphological and/or genetic data: *L.nkongsamba***sp. nov.** from the Nlonako Ecological Reserve (1000–1400 m asl) in the sub-montane zone and *L.yabassi***sp. nov**. from Yabassi in the lowlands. A redescription and amended diagnostic features of *L.edeaensis* and *L.balssi* are provided, and the genus diagnosis is updated to accommodate all four species. An identification key is also provided for the species of *Louisea*. A tree of phylogenetic relationships based on three mtDNA loci (COI, 12S rRNA, and 16S rRNA) supports the taxonomic revision, and indicates speciation of *Louisea* species along an altitudinal gradient, but further phylogenetic analyses are needed to understand whether this can lend support to the hypothesis that there is a montane centre of speciation along the Cameroon Volcanic Line. The phylogenetic tree also shows that *Buea* Cumberlidge, Mvogo Ndongo, Clark & Daniels, 2019 and *Potamonemus* Cumberlidge & Clark, 1992 are sister genera that may be derived from the *Louisea* lineage.

## Introduction

The freshwater crab genus *Louisea* Cumberlidge, 1994, was established by Cumberlidge (1994) to accommodate two species from Cameroon. The first of these, *L.edeaensis* (Bott, 1969), was originally described by Bott (1969) as *Globonautesmacropusedeaensis* Bott, 1969 from a single male specimen from Edea, Cameroon collected in 1910. The second species is *L.balssi* (Bott, 1959), which was described by Bott (1959) as *Globonautesbalssi* based on specimens from Kumba, Cameroon collected between 1900 and 1910. This taxon was later treated by Bott (1970) as the subspecies *Globonautesmacropusbalssi* (Bott, 1970). Cumberlidge (1999) found that the genus *Globonautes* Bott, 1959, is endemic to the Upper Guinea Forests from Liberia to Guinea, and that *Louisea* is endemic to the Lower Guinea Forests in southwestern Cameroon. Both *L.edeaensis* and *L.balssi* were assessed as endangered (EN) under the International Union for Conservation of Nature (IUCN) Red List protocols, and it was thought at that time that both these species might even be extinct (IUCN 2003, Cumberlidge 2008a, 2008b, Cumberlidge et al. 2009). However, our recent biotic surveys in Cameroon (in August 2015 and May 2017) have led to the rediscovery of *L.edeaensis* from lowland forests below 400 m asl (Bedimet Island of the Lake Ossa wetland complex), and of *L.balssi* from high altitude forests above 1300 m asl (Mt. Manengouba) (Mvogo Ndongo et al. 2017a, 2018). The molecular data from the fresh specimens established the validity of the genus *Louisea*, and enabled the resolution of longstanding questions surrounding the phylogenetic relationships of the *Louisea* that was definitively assigned to the subfamily Potamonautinae Bott, 1970 (Mvogo Ndongo et al. 2017c).

The rediscoveries of *L.edeaensis* and *L.balssi* prompted renewed extensive systematic surveys of the lowland and upland zones in the tropical rainforests of southwestern Cameroon in 2017 and 2018, which resulted in the recognition of a new species of *Louisea* from the submontane zone of Mt. Nlonako (1000–1400 m asl). This new species is described here based on morphological and genetic data. A second new species of *Louisea* is also recognised from Yabassi in the lowlands of southwestern Cameroon based on the re-examined specimens from the Museum für Naturkunde, Berlin, Germany (ZMB) that had previously been identified as belonging to *Louisea* (Cumberlidge 1994, 1999, Mvogo Ndongo et al. 2017a). The genus *Louisea* is revised here to include four species, *L.edeaensis* is redescribed, and the diagnosis of *L.balssi* is amended. The taxonomic revision of *Louisea* is based on a unique combination of characters of the carapace, thoracic sternum, chelipeds, and male first gonopods. It is also supported by original data on the phylogenetic relationships of *Louisea* and the other species of freshwater crabs found during our surveys of the rainforest ecosystems of southwestern Cameroon. The present molecular study used three mitochondrial genes (COI, 12S rRNA, and 16S rRNA) but included only three of the four species of *Louisea* (because no fresh specimens of the new species from Yabassi could be collected). Also included in the phylogenetic tree and species of the two other genera found in southwestern Cameroon: *Buea* Cumberlidge, Mvogo Ndongo, Clark & Daniels, 2019 and *Potamonemus* Cumberlidge & Clark, 1992.

## Materials and methods

### Sampling

**Figure 1.**
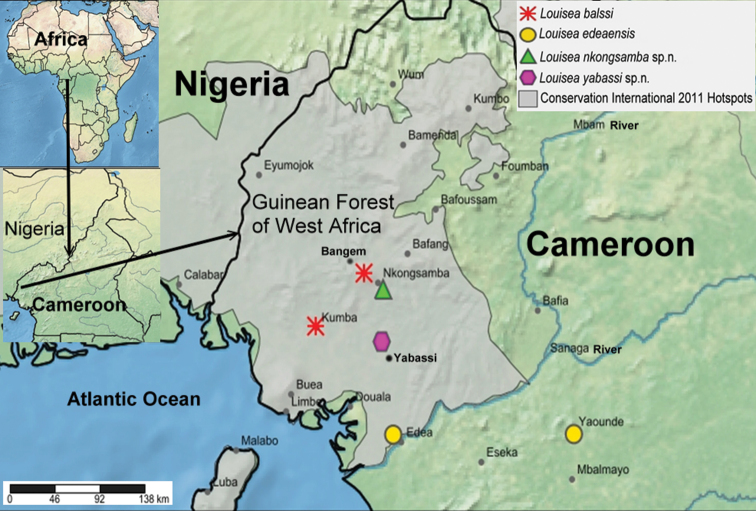
Collection localities of the four species of *Louisea* in Cameroon.

A series of field surveys of freshwater decapods undertaken in southwestern Cameroon from 2015 to 2018 focused on biodiversity hotspots that had been previously identified in 2011 by Conservation International for other freshwater taxa (Fig. 1). Crabs were hand-caught from puddles near small permanent streams, from under fallen leaves, and from their burrows sited close to water bodies. Specimens of *Louisea* sp. (ZMB Crust. 21575) collected from Yabassi, Cameroon between 1900 and 1910 held in the Museum für Naturkunde were re-examined here.

### Morphological analyses

All measurements (in mm) were taken with digital callipers. The terminology used follows Cumberlidge (1999), and the classification follows Ng et al. (2008). Characters of the gonopods, carapace, thoracic sternum, chelipeds, third maxillipeds, and mandibles were examined in detail, and photographs were taken using a Leica microscope (model Z16A POA), and LAS V4 and Helicon Focus 6.7.1 software. Post processing of the images was undertaken using Adobe Photoshop CC5. The type specimens of the two new species and the freshly collected specimens of *Louisea* species are deposited in the Museum für Naturkunde, Berlin, Germany (**ZMB**). Other material is deposited in the Institute of Fisheries and Aquatic Sciences, University of Douala at Yabassi (**IFAS**), the Senckenberg Museum, Frankfurt, Germany (**SMF**), the Zoological Institute Museum, Hamburg, Germany (**ZIM**), the Naturhistorisches Museum Wien, Austria (**NHMW**), and the Zoologische Sammlung des Bayerischen Staates, München, Germany (**ZSBS**).

The following abbreviations are used:

a pleonal (abdominal) segment or pleomere;

a5/a6 sulci between adjacent pleomeres;

asl above sea level;

CW carapace width measured at widest point;

CL carapace length measured along medial line from anterior to posterior margin;

CH carapace height measured at maximum height of cephalothorax;

e episternite;

FW front width measured along anterior frontal margin between inner angles of orbits;

G1 male first gonopod;

G2 male second gonopod;

p2–p5 pereiopods 2–5 or walking legs 1–4;

SS subterminal segment of G1 or G2;

s4/e4 (s4/e4, s5/e5, s6/e6, s7/e7) episternal sulci between adjacent thoracic sternites and episternites;

s thoracic sternite;

s1/s2 (s1/s2, s2/s3, s4/s5, s5/s6, s6/s7) sternal sulci between adjacent thoracic sternites;

TA terminal article of G1 or G2;

TS terminal segment of mandibular palp.

### Molecular analysis

Genomic DNA was extracted from a tissue sample of up to 25 mg cut from the pereiopod muscle of 70% ethanol-preserved specimens using the Qiagen DNeasy Blood & Tissue kit.

Polymerase chain reaction (PCR) was used to amplify three mitochondrial gene fragments, a ~ 638 bp region of the 16S ribosomal RNA gene (16S) using primers 16L29 and 16HLeu or 16H10 (Schubart 2009), a ~ 594 bp region of the 12S ribosomal RNA gene (12S) using primers 12L4 and 12H2 (Schubart 2009), and a 648 bp fragment of the Cytochrome Oxidase subunit I gene (COI) using primers COL6a (Schubart 2009) and COH1b (Schubart 2009), COH6 (Schubart and Huber 2006), or CO1a (Palumbi et al. 1991). PCR was performed in 25 μl volumes containing 1× Taq buffer, 1.5 mM MgCl_2_, 200 μM each dNTP, 1 U Taq polymerase, ca. 50–100 ng DNA and ddH_2_O up to volume. After an initial denaturation step of 4 min at 94 °C, cycling conditions were 35 cycles at 94 °C for 30 s, 45 °C for 60 s, and 72 °C for 90 s, with a final elongation step of 5 min at 72 °C. The same primers were used in PCR and sequencing.

PCR products were sent to Macrogen Europe for purification and cycle sequencing of both strands of each gene. The sequences obtained were proofread manually using Chromas and aligned with Bioedit. Results from these genes were concatenated into a single alignment that was then converted into a Nexus file with FaBox (Villesen 2007). The best evolutionary model was determined with jModeltest v.2.1.7 (Darriba et al. 2012) based on the Akaike information criterion (Posada and Buckley 2004) and resulted in the GTR+I+G (COI), GTR+G (16S) and HKY+G (12S) models. The phylogenetic reconstruction was conducted with Maximum Likelihood (ML) using the software RAxML (Stamatakis 2006) under GTR + (I) + G model of sequence evolution. Bayesian Inference (BI) was performed to infer phylogeny by using MrBayes v. 3.2.2 (Huelsenbeck and Ronquist 2001). The MCMC was run with four independent chains for 10,000,000 generations, samplefreq = 500, and burnin = 10,001. Analyses were conducted separately to test for topology congruence. The trees were drawn to scale, with branch lengths measured as the number of substitutions. All sequences generated for this study have been uploaded to GenBank (Table 1).

**Table 1.** Species of *Louisea*, *Buea*, *Potamonemus* and outgroups included in the molecular analyses. The newly-presented data are given in bold.

**Species**	**Locality**	**Museum number**	**Reference study**	**GenBank accession number**		
				**CO1**	**12S rRNA**	**16S rRNA**
***L.nkongsamba* sp. nov.**	Nlonako	ZMB Crust. 31618	Present	MN188072	MN217386	MN217393
***L.nkongsamba* sp. nov.**	Nlonako	ZMB Crust. 31620	Present	MN188065	MN217387	MN217394
***L.balssi* (CW 16.2 mm)**	Manengouba	ZMB Crust. 30319	Present	MN188071	MN217385	MN217392
***L.balssi* (CW 14.8 mm)**	Manengouba	ZMB Crust.29628	Present	MN188070	MN217384	MN217391
*L.edeaensis* (CW 17.5 mm)	Lake Ossa	LZUY 15-3 (T351-30)	Mvogo Ndongo et al. 2017c	KY964474	KY964479	KY964472
***L.edeaensis* (CW 16.15 mm)**	Lake Ossa	ZMB Crust. 30335	Present	MN188068	–	MN217395
***Buea* sp.1**	N.P. Korup	ZMB Crust. 30321	Present	MN188069	MN217388	MN217396
***Buea* sp.2**	N.P. Bakossi	ZMB Crust. 30325	Present	MN188066	MN217389	MN217397
*B.asylos*	Buea and Kumba	NHM 1994.588-591	Daniels et al. 2015	KP640489	KP640410	KP640453
***Potamonemus* sp.**	N.P. Bakossi	ZMB Crust. 30327	Present	MN188067	MN217390	MN217398
*P.mambilorum*	southwest Cameroon	NHM 1991.183	Daniels et al. 2015	–	KP640409	KP640452
*P.sachsi*	southwest Cameroon	NMU09.04.1983	Daniels et al. 2002	–	AY803490	AY803530
*Afrithelphusamonodosa*	Guinea	NMU 25.IV.2005.C	Daniels et al. 2015	KP640469	KP640386	KP640430
*Globonautesmacropus*	Guinea	NMU VII. 1988	Daniels et al. 2015	–	KP640391	KP640435

LZUY: Zoological Collection of the Laboratory of Zoology, University of Yaounde 1, Cameroon; NHM: Natural History Museum, London, UK; NMU: Northern Michigan University Museum, USA; ZMB: Museum für Naturkunde, Berlin, Germany.

## Systematic accounts

### Infraorder Brachyura Latreille, 1802

#### Superfamily Potamoidea Ortmann, 1896

**Family Potamonautidae Bott, 1970**

**Subfamily Potamonautinae Bott, 1970**

##### Genus Louisea

Taxon classificationAnimaliaDecapodaPotamonautidae

Cumberlidge, 1994

D44FD7D8-9485-5BB4-867F-140EADCA8612

Globonautes Bott 1959: 995, pl. 1, figs 1–6; 1969: 359; 1970: 23.
Louisea Cumberlidge 1994: 123; 1999: 226; Ng et al. 2008: 169 (list).

###### Type species.

*Globonautesmacropusedeaensis* Bott, 1969, by original designation; gender feminine.

###### Diagnosis.

**Figure 2.**
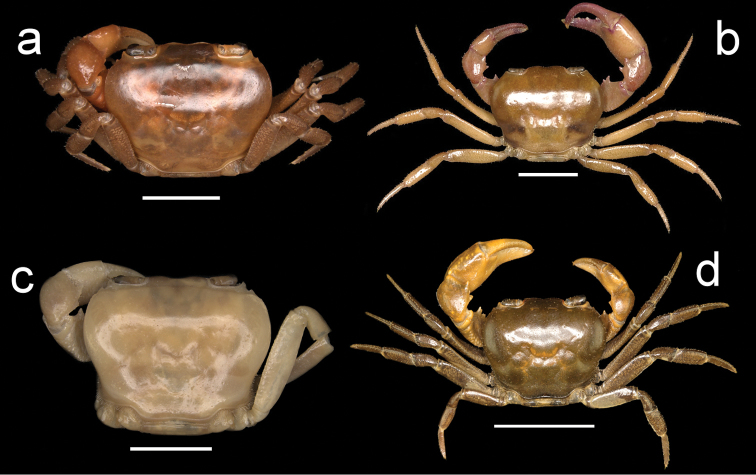
Four species of *Louisea* endemic to southwestern Cameroon, whole animal, dorsal view. **a** Largest adult male (CW 17.5 mm) of *L.edeaensis* from Lake Ossa wetland complex (ZMB Crust. 26930) (missing right cheliped) **b** largest adult male (CW 16.2 mm) of *L.balssi* from Man’s Crater Lake Manengouba (ZMB Crust. 30319) (missing left p5) (‘Mvogo Ndongo et al. 2018: fig. 1’, www.mapress.com/j/zt) **c** largest adult male, holotype (CW 18.1 mm) of *L.yabassi* sp. nov. from Yabassi (ZMB Crust. 21575) (missing left p2–p5, and right cheliped and p2, p3) **d** largest adult male, holotype (CW 20.0 mm) of *L.nkongsamba* sp. nov. from Mt. Nlonako (ZMB Crust. 31618). Scale bars: 8 mm (**a**), 9 mm (**b**), 8.30 mm (**c**), 17 mm (**d**).

**Figure 3.**
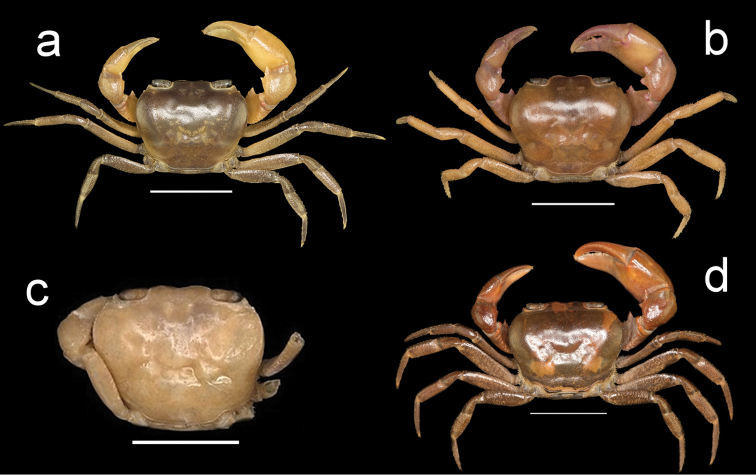
Four species of *Louisea* endemic to southwestern Cameroon, whole animal, dorsal view. **a** Second largest adult male (CW 16.1 mm) of *L.edeaensis* from Lake Ossa wetland complex (ZMB Crust. 30335) (missing left p4) **b** second largest adult male (CW 14.8 mm) of *L.balssi* from Man’s Crater Lake Manengouba (ZMB Crust. 30319) (missing left p4 and right p2) **c** subadult male, paratype (CW 13.8 mm) of *L.yabassi* sp. nov. from Yabassi (ZMB Crust. 21575) (missing left p2–p4, and right cheliped and p2–p5) **d** second largest adult male (CW 18.38 mm) of *L.nkongsamba* sp. nov. from Mt. Nlonako (ZMB Crust. 31618). Scale bars: 12.42 mm (**a**), 10 mm (**b**), 8.42 mm (**c**), 11.63 mm (**d**).

**Figure 4.**
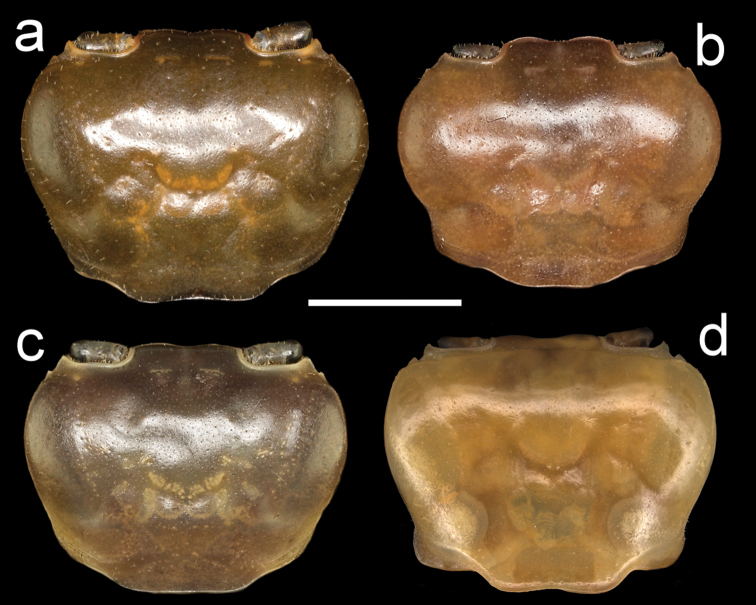
Four species of *Louisea* endemic to southwestern Cameroon, carapace, dorsal view. **a** Largest adult male, holotype (CW 20.0 mm) of *L.nkongsamba* sp. nov. from Mt. Nlonako (ZMB Crust. 31618) **b** second largest adult male (CW 14.8 mm) of *L.balssi* from Man’s Crater Lake Manengouba (ZMB Crust. 30319) **c** second largest adult male (CW 16.1 mm) of *L.edeaensis* from Lake Ossa wetland complex (ZMB Crust. 30335) **d** adult male, holotype (CW 18.1 mm) of *L.yabassi* sp. nov. from Yabassi (ZMB Crust. 21575). Scale bars: 9.1 mm (**a**), 7.25 mm (**b**), 7.90 mm (**c**), 8.62 mm (**d**).

Amended from Cumberlidge (1994, 1999). Carapace ovoid, high (CH/FW 1.28–2.12, *N* = 57) with faint urogastric groove (Figs 2a–d, 3a–d). Postfrontal crest detectable (either prominent or faint), but meeting anterolateral margins of carapace (Fig. 7a–d). Exorbital, intermediate teeth small, but detectable; epibranchial tooth minute, almost undetectable (Figs 4, 7a–d). Medial inferior margin of merus of cheliped with large jagged tooth one-third from distal margin, followed by numerous distinct smaller teeth decreasing in size proximally (Fig. 9a–d). Third maxilliped exopod completely lacking flagellum; third maxilliped ischium with vertical groove (Fig. 14a–d). Mandibular palp 2 segmented; terminal segment (TS) bilobed, with large anterior lobe (0.5–0.8 × TS length) (Fig. 15a–d). G1 highly stout, distinctly sinuous; terminal article (TA) short, about one quarter length of subterminal segment (SS) (TA/SS 0.22–0.29), directed outwards at 45° angle to longitudinal axis of G1; SS, inverted funnel-shaped, proximally distinctly broad, abruptly narrow, slim, distal two-thirds tube-like; G1SS stout, tapering slightly from wide basal margin to relatively wide distal margin (0.6 × SS basal margin), dorsal face with broad dorsal membrane (maximum width 0.1 × SS length) at TA/SS junction (Figs 11a–d, 12a–d). G2TA long (TA/SS 0.40–0.44), flagellum-like, almost as long as G2SS (Fig. 13a–d). Small species (CW 14–22 mm in adults).

###### Distribution.

*Louisea* is endemic to southern Cameroon (Cumberlidge 1994, 1999) (Fig. 1). *Louiseaedeaensis* is known from Yaounde, Edea, and the Lake Ossa faunal reserve, while *L.balssi* is known from the Bakossi region at Barombi Mbo near Kumba and from Manengouba Ecological Reserve (Cumberlidge 1994, 1999, Mvogo Ndongo et al. 2017a, 2018). *Louiseankongsamba* sp. nov. is known from Mt. Nlonako Ecological Reserve at Nkongsamba, while *Louiseayabassi* sp. nov. is from Yabassi.

###### Remarks.

Cumberlidge (1994, 1999) provided the diagnostic characters of *Louisea* based mainly on the male holotype of *L.edeaensis*, because *L.balssi* was only known then from a juvenile male. The revision of the diagnostic characters for this genus is based on our examinations of adult males of all four species included here (*L.edeaensis*, *L.balssi*, *L.nkongsamba* sp. nov., and *L.yabassi* sp. nov.). The amended character descriptions are also used to compare *Louisea* with other potamonautid genera.

The bilobed terminal segment of the mandibular palp of *Louisea* is unusual, and it sets this genus apart from most genera in the Potamonautinae that typically possess a simple mandibular palp (i.e., with no additional anterior lobe) (Cumberlidge 1999; Cumberlidge et al. 1999; Cumberlidge and Reed 2003). This is true for *Erimetopus* Rathbun, 1894 and *Platythelphusa* A. Milne-Edwards, 1887, and all but one species of *Potamonautes* MacLeay, 1838 [*P.brincki* (Bott, 1960) being the exception], most species of *Sudanonautes* Bott, 1955 [except for *S.floweri* (de Man, 1901) and *S.orthostylis* Bott, 1955], and most species of *Liberonautes* Bott, 1955 (Cumberlidge 1999). It should be noted that in those potamonautine species without a simple mandibular palp, the anterior lobe is little more than a hard ledge at the junction between the segments, rather than a true lobe shape (Cumberlidge 1999). The terminal segment of the mandibular palp of *Louisea* is distinctly bilobed (with an anterior lobe 0.6 × the terminal segment length) and is superficially similar to the mandibular palps of *Afrithelphusa* Bott, 1969 and *Globonautes* (the two West African genera assigned to the Deckeniinae Ortmann, 1897, but the mandibular palp of the each of the latter two genera has a larger anterior lobe that is subequal to the posterior lobe (Fig. 15a–d; Cumberlidge 1999: fig. 48A–C).

The lack of a flagellum on the exopod of the third maxilliped in *Louisea* is rarely seen in other species of the Potamonautinae, and most of the members of this subfamily typically possess a long flagellum on the third maxilliped exopod (Cumberlidge 1999). The exceptions to this are the species of *Buea* and *Potamonemus*, and *Liberonautesgrandbassa* Cumberlidge, 1999 and *L.lugbe* Cumberlidge, 1999 (Cumberlidge and Clark 1992; Cumberlidge 1993, 1999; Cumberlidge et al. 2019). The lack of a flagellum on the exopod of the third maxilliped of *Louisea* is also shared with species of the Deckeniinae (*Afrithelphusa*, *Globonautes*, and *Madagapotamonhumberti* Bott, 1965) (Cumberlidge 1999; Cumberlidge et al. 2008). *Louisea* can also be distinguished from the West African Deckeniinae genera *Afrithelphusa* and *Globonautes* by characters of the gonopods (G1TA shape and G2TA length), the presence or absence of an intermediate tooth between the exorbital and epibranchial teeth (Cumberlidge 1999), and by molecular evidence (Daniels et al. 2015; Mvogo Ndongo et al. 2017c).

##### Louisea edeaensis

Taxon classificationAnimaliaDecapodaPotamonautidae

(Bott, 1969)

753C9217-7DAB-5B4E-83A2-B578150518A8

Figs 2a , 3a , 4c , 5a , 6a , 7a , 8a, b , 9a , 10a , 11a , 12a , 13a , 14a , 15a

Globonautes macropus edeaensis Bott 1969: 360; 1970: 24, pl. 1, figs 3–5, pl. 26, fig. 8; Cumberlidge 1987: 2215, table 2.
Louisea edeaensis Cumberlidge 1994: 124, fig. 1, table 1; 1997: 577; 1999: 227, 5300, 54–57, figs 46F, 47E, 48E, 49F, 51F, 52F, 53DD, 54–57, 62F, 68F, table IX, plate 3; Ng et al. 2008: 169 (list); Cumberlidge et al. 2009: 6; Mvogo Ndongo et al. 2017a: 273, figs 1–3; 2017c: 440, fig. 1.

###### Material examined.

CAMEROON. Holotype: adult male (CW 22.5 mm), Edea, Jan 1910, coll. Riggenbach (ZSBS 1118/1). Adult male, 2 adult females (CWs 19.4, 17.5 mm), Yaounde, 1907, coll. Haberer (NHMW 1877). See Table 2 for details of the material examined from Lake Ossa.

**Table 2.** Morphometric analysis and collection data of specimens (*N* = 22) of *Louiseaedeaensis* from Cameroon (Lake Ossa; 3°48'56.1"N, 10°03'18.5"E; 90 m a.s.l.). All measurements are given in mm.

**Specimens**	**CW**	**CL**	**CH**	**FW**	**CW/FW**	**CL/FW**	**CH/FW**	**FW/CL**	**Coll. Date**	**Museum**
1 ad♂	17.5	13.5	6.8	5.8	3.01	2.32	1.17	0.43	P.A.M.N 10.07. 15	ZMB Crust. 26930
2 ad♂	16.15	12.70	7.80	5.10	3.17	2.49	1.53	0.40	P.A.M.N 15.01. 16	ZMB Crust. 30335
3 ad♂	15.60	13.1	7.90	4.80	3.25	2.73	1.64	0.37	P.A.M.N 15.01. 16	IFAS-001
4 ad♂	14	11.03	6.5	4.5	3.11	2.51	1.44	0.39	P.A.M.N 15.01. 16	ZMB Crust. 30335
5 ad♂	15.35	12.15	8.30	5	3.07	2.43	1.66	0.41	P.A.M.N 10.07. 15	ZMB Crust. 30319
6 ad♀	19.90	15.2	7.7	5.9	3.37	2.57	1.30	0.38	P.A.M.N 10.07. 15	LZUY 15-2 (IFAS-002)
7 ad ♀	17.5	13.30	6.6	5.3	3.30	2.50	1.24	0.39	P.A.M.N 11.11. 16	LZUY 15-2 (IFAS-002)
8 ad♀	17.0	13.2	6.30	5.2	3.26	2.53	1.21	0.39	P.A.M.N 11.11. 16	LZUY 15-2 (IFAS-002)
9 ad♀	14.80	11.30	7.2	4.9	3.02	2.30	1.46	0.43	P.A.M.N 15.01. 16	LZUY 15-3 (T351-30)
10 ad ♀	17.30	13.80	9.80	5.90	2.90	2.33	1.66	0.42	P.A.M.N 10.07. 15	LZUY 15-2 (IFAS-002)
11 ad♀	14.6	11.2	7.1	4.90	2.97	2.28	1.44	0.43	P.A.M.N 15.01. 16	LZUY 15-1 (IFAS-003)
12 ad ♀	18.90	13.89	10.50	6.01	3.14	2.31	1.74	0.43	P.A.M.N 15.01. 16	ZMB Crust. 30335
13 ad♀	14.10	11.20	7	4.7	3.0	2.38	1.48	0.41	P.A.M.N 10.07. 15	IFAS-004
14 sd♀	13	10.50	5.80	4	3.25	2.62	1.45	0.38	P.A.M.N 10.07. 15	LZUY 15-3 (IFAS-005)
15 sd♂	11.80	10.09	5.80	4	2.95	2.52	1.45	0.39	P.A.M.N 11.11. 16	IFAS-004
16 sd♂	11.70	9.89	6	4	2.92	2.47	1.5	0.40	P.A.M.N 11.11. 16	IFAS-004
17 sd♂	12.40	9.80	5.7	4	3.1	2.45	1.42	0.40	P.A.M.N 11.11. 16	IFAS-004
18 sd♂	12.00	9.5	5.6	4	3	2.37	1.4	0.42	P.A.M.N 10.07. 15	LZUY 15-1 (IFAS-003)
19 sd♀	13.60	10.01	6.80	4.15	3.27	2.41	1.63	0.41	P.A.M.N 11.11. 16	IFAS-004
20 sd♀	12.80	9.8	6	4	3.2	2.45	1.5	0.40	P.A.M.N 11.11. 16	IFAS-004
21 sd♀	11.60	10	5.5	3.8	3.05	2.63	1.44	0.38	P.A.M.N 11.11. 16	IFAS-004
22 sd♀	11.01	9.50	5.40	3.8	2.89	2.5	1.42	0.40	P.A.M.N 11.11. 16	LZUY 15-4 (IFAS-005)
**Mean**	**14.02**	**11.07**	**6.36**	**4.51**	**2.96**	**2.45**	**1.41**	**0.40**	–	–

Key: P.A.M.N: Pierre A. Mvogo Ndongo; ad: adult; sd: subadult.

###### Diagnosis.

Amended from Cumberlidge (1994, 1999); Mvogo Ndongo et al. (2017a). Carapace smooth, urogastric groove faint (Figs 2a, 3a, 4c). Postfrontal crest faint, complete, meeting anterolateral margin behind intermediate tooth (Fig. 7a). Exorbital, intermediate teeth small, low, distinct (Figs 2a, 3a, 4c, 7a). Third maxilliped ischium with distinct vertical groove (Fig. 14a). Terminal segment (TS) of mandibular palp bilobed, with large distinct anterior lobe (0.6 × terminal segment length) (Fig. 15a). Major cheliped dactylus relatively stout, straight, with two large teeth (one proximal, one medial) (Fig. 8a); cheliped propodus with four large teeth (three proximal, one distal); cheliped carpus inner margin with long, broad distal tooth followed by slim, smaller proximal tooth (Fig. 10a). G1TA short (TA/SS = 0.3), directed outward at 45° angle to longitudinal axis of G1SS, proximally broad, distal two-thirds narrowing abruptly to form slim tube (Figs 11a, 12a). G1SS tapering slightly from broad basal margin to relatively wide distal margin (0.6 × SS basal margin), dorsal face with broad dorsal membrane (maximum width 0.1 × SS length) at TA/SS junction (Fig. 11a). G2TA long (TA/SS = 0.40), flagellum-like, almost as long as G2SS (Fig. 13a). Mature between CWs 14–23 mm.

###### Re-description.

**Figure 5.**
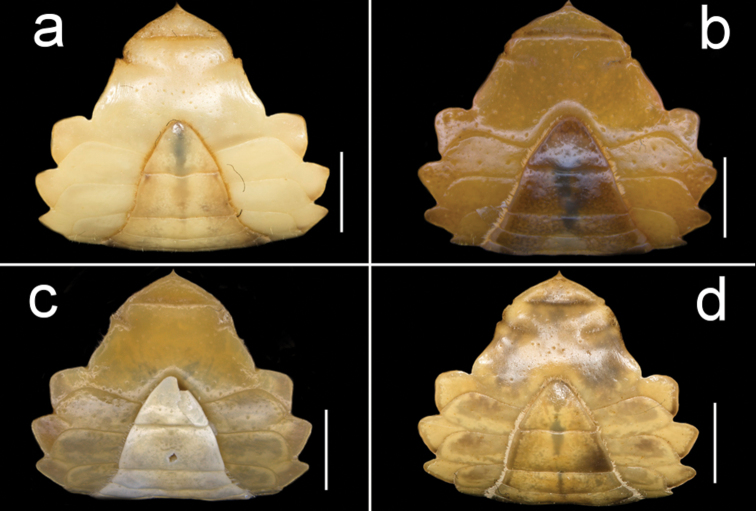
Four species of *Louisea* endemic to southwestern Cameroon, thoracic sternites (s1–s8) and pleonal segments (a4–a7). **a** Second largest adult male (CW 16.1 mm) of *L.edeaensis* from Lake Ossa wetland complex (ZMB Crust. 30335) **b** largest adult male (CW 16.2 mm) of *L.balssi* from Man’s Crater Lake Manengouba (ZMB Crust. 30319) (missing left p5) (‘Mvogo Ndongo et al. 2018: fig. 2c’, www.mapress.com/j/zt) **c** adult male, holotype (CW 18.1 mm) of *L.yabassi* sp. nov. from Yabassi (ZMB Crust. 21575) **d** largest adult male, holotype (CW 20.0 mm) of *L.nkongsamba* sp. nov. from Mt. Nlonako (ZMB Crust. 31618). Scale bars: 12.42 mm (**a**), 9 mm (**b**), 8.30 mm (**c**), 17 mm (**d**).

**Figure 6.**
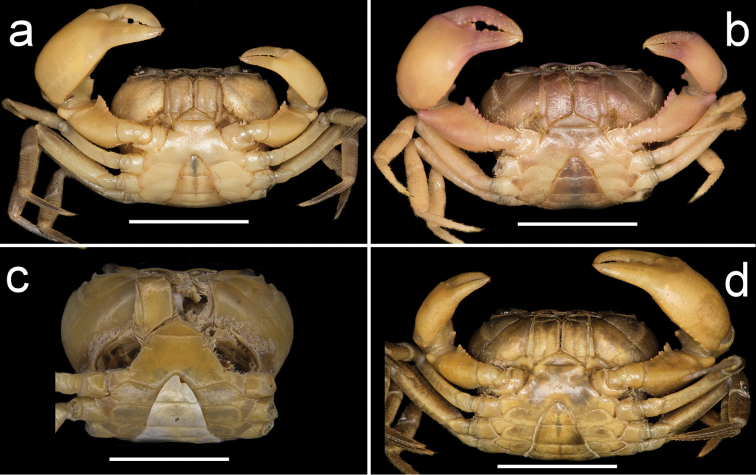
Four species of *Louisea* endemic to southwestern Cameroon, whole animal, ventral view. **a** Second largest adult male (CW 16.1 mm) of *L.edeaensis* from Lake Ossa wetland complex (ZMB Crust. 30335) **b** second largest adult male (CW 14.8 mm) of *L.balssi* from Man’s Crater Lake Manengouba (ZMB Crust. 30319) **c** adult male, holotype (CW 18.1 mm) of *L.yabassi* sp. nov. from Yabassi (ZMB Crust. 21575) **d** largest adult male, holotype (CW 20.0 mm) of *L.nkongsamba* sp. nov. from Mt. Nlonako (ZMB Crust. 31618). Scale bars: 11.63 mm (**a**), 9.61 mm (**b**), 9.50 mm (**c**), 11.60 mm (**d**).

**Figure 7.**
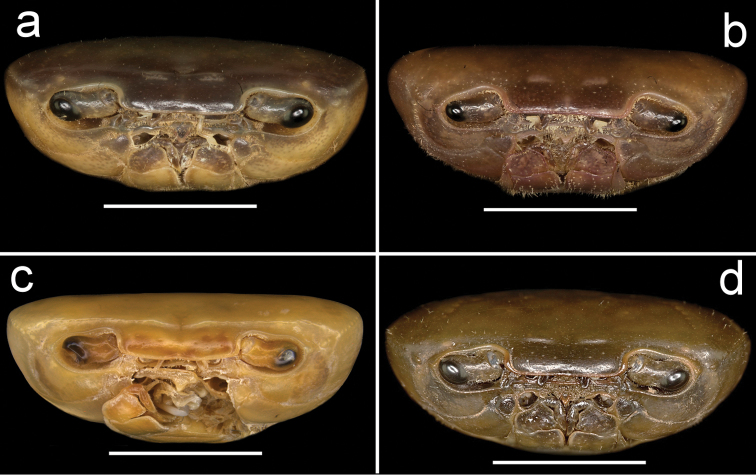
Four species of *Louisea* endemic to Southwestern Cameroon, carapace, frontal view. **a** Second largest adult male (CW 16.1 mm) of *L.edeaensis* from Lake Ossa wetland complex (ZMB Crust. 30335) **b** second largest adult male (CW 14.8 mm) of *L.balssi* from Man’s Crater Lake Manengouba (ZMB Crust. 30319) **c** adult male, holotype (CW 18.1 mm) of *L.yabassi* sp. nov. from Yabassi (ZMB Crust. 21575) **d** largest adult male, holotype (CW 20.0 mm) of *L.nkongsamba* sp. nov. from Mt. Nlonako (ZMB Crust. 31618). Scale bars: 7.20 mm (**a**), 6.60 mm (**b**), 8.06 mm (**c**), 8.70 mm (**d**).

Amended from Cumberlidge (1994, 1999). Carapace ovoid, high, slightly arched (CH/FW 1.41, *N* = 22), wide (CW/FW 3.14, *N* = 22), smooth; postfrontal crest poorly defined, completely crossing carapace, meeting anterolateral margins of carapace behind intermediate tooth (Fig. 7a); mid-groove broad, shallow, epigastric crests poorly defined (Fig. 7a); external orbital tooth small, low; epibranchial tooth reduced to granule, almost undetectable (Figs 4a, 7a); intermediate tooth on anterolateral margin between external orbital, epibranchial teeth small but detectable (Figs 4a, 7a); anterolateral margin lined by small granules (Figs 4a, 7a); posterolateral margin continuous with anterolateral margin, curving inward; posterior margin of carapace wide (2/3 CW). Carapace branchiostegal wall with 2 sutures dividing it into 3 parts (Fig. 6a); longitudinal suture beginning at respiratory opening dividing suborbital, subhepatic regions from pterygostomial region (Fig. 6a); vertical suture beginning at epibranchial tooth curving sharply down to meet longitudinal suture, marked by row of granules (Figs 6a, 7a). Sternal sulcus s2/s3, deep, complete, s3 lacking depression (Fig. 5a); s3/s4 reduced to 2 short, distinct notches on each side of sternum (Fig. 5a); episternal sulci s4/e4, s5/e5, s6/e6 faint or missing, s7/e7 complete (Fig. 5a). Mandibular palp 2 segmented; terminal segment (TS) bilobed, anterior lobe 0.6 × length of terminal segment (Fig. 15a, d). Third maxillipeds (Fig. 6a) filling entire buccal cavern, except for transversely oval efferent respiratory openings in superior lateral corners; ischium with distinct vertical groove (Fig. 14a); exopod lacking flagellum (Fig. 14a).

**Figure 8.**
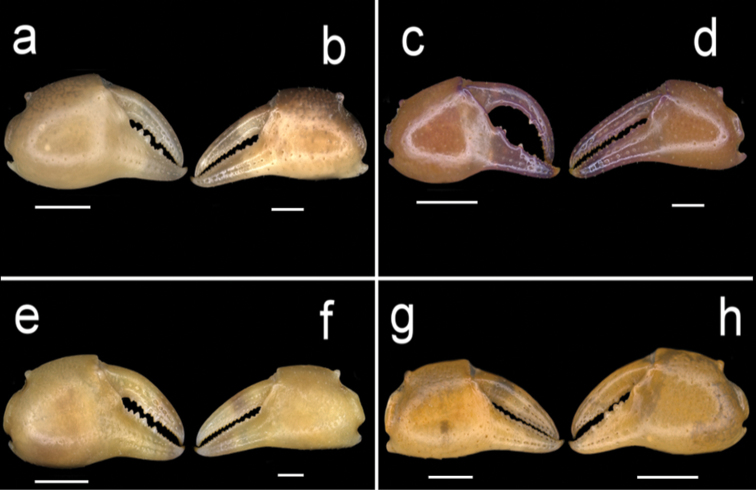
Four species of *Louisea* endemic to Southwestern Cameroon, frontal view of right and left chela. **a, b** Second largest adult male (CW 16.1 mm) of *L.edeaensis* from Lake Ossa wetland complex (ZMB Crust. 30335) **c, d** largest adult male (CW 16.2 mm) of *L.balssi* from Man’s Crater Lake Manengouba (ZMB Crust. 30319) (missing left p5) (‘Mvogo Ndongo et al. 2018: fig. 3a, b’, www.mapress.com/j/zt) **e, f** adult male, holotype (CW 18.1 mm) of *L.yabassi* sp. nov. from Yabassi (ZMB Crust. 21575) **g, h** largest adult male, holotype (CW 20.0 mm) of *L.nkongsamba* sp. nov. from Mt. Nlonako (ZMB Crust. 31618). Scale bars: 5 mm (**a, c, e, g**), 2 mm (**b, d, f**), 10 mm (**h**).

**Figure 9.**
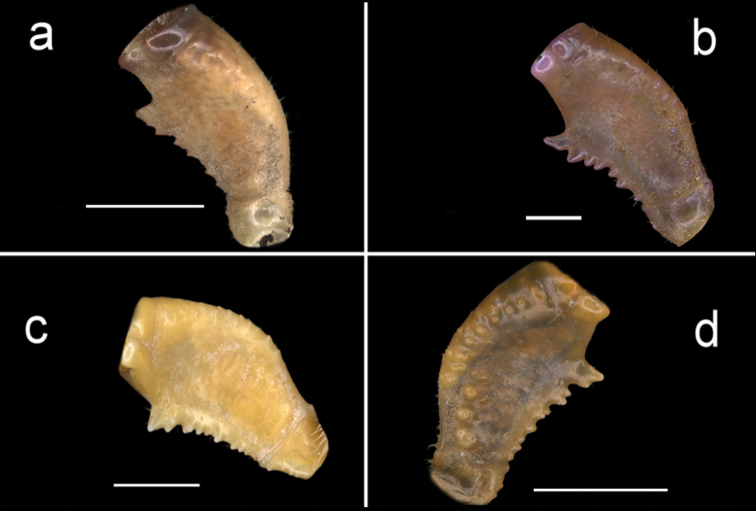
Four species of *Louisea* endemic to southwestern Cameroon, right cheliped merus. **a** Second largest adult male (CW 16.1 mm) of *L.edeaensis* from Lake Ossa wetland complex (ZMB Crust. 30335) **b** largest adult male (CW 16.2 mm) of *L.balssi* from Man’s Crater Lake Manengouba (ZMB Crust. 30319) (missing left p5) (‘Mvogo Ndongo et al. 2018: fig. 3d’, www.mapress.com/j/zt) **c** adult male, holotype (CW 18.1 mm) of *L.yabassi* sp. nov. from Yabassi (ZMB Crust. 21575) **d** largest adult male, holotype (CW 20.0 mm) of *L.nkongsamba* sp. nov. from Mt. Nlonako (ZMB Crust. 31618). Scale bars: 5 mm (**a, c**), 2 mm (**b**), 10 mm (**d**).

**Figure 10.**
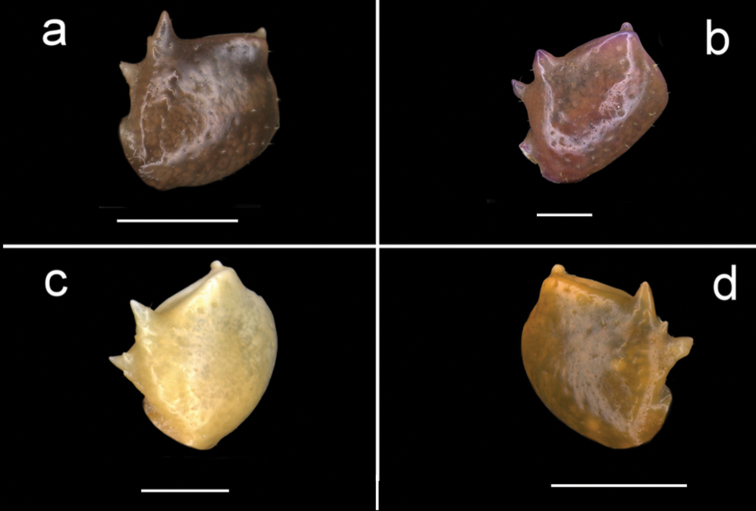
Four species of *Louisea* endemic to southwestern Cameroon, right cheliped carpus. **a** Second largest adult male (CW 16.1 mm) of *L.edeaensis* from Lake Ossa wetland complex (ZMB Crust. 30335) **b** largest adult male (CW 16.2 mm) of *L.balssi* from Man’s Crater Lake Manengouba (ZMB Crust. 30319) (‘Mvogo Ndongo et al. 2018: fig. 3c’, www.mapress.com/j/zt) **c** adult male, holotype (CW 18.1 mm) of *L.yabassi* sp. nov. from Yabassi (ZMB Crust. 21575) **d** largest adult male, holotype (CW 20.0 mm) of *L.nkongsamba* sp. nov. from Mt. Nlonako (ZMB Crust. 31618). Scale bars: 5 mm (**a, c**), 2 mm (**b**), 10 mm (**d**).

Male chelipeds greatly unequal, right cheliped larger than left cheliped (Figs 3a, 6a). Dactylus, propodus of right (major) cheliped slim, elongated; fixed finger (propodus) with 4 large pointed teeth (3 proximal, 1 distal); movable finger (dactylus) relatively stout, straight, with 2 large teeth (1 proximal, 1 medial) (Fig. 8a). Dactylus, propodus of left (minor) cheliped slender, with small teeth on occluding margins (Figs 2a, 3a). Medial inferior margin of cheliped merus with large jagged distal tooth angled outward at 90°, followed by numerous distinct smaller teeth decreasing in size proximally (Figs 6a, 9a; see Mvogo Ndongo et al. 2017a: fig. 1B). Cheliped carpus inner margin with 2 large pointed teeth, distal tooth long, broad with pointed tip; proximal tooth longer than distal tooth (Fig. 10a). Walking legs (p2–p5) slender, p4 longest, p5 shortest; dactyli (p2–p5) tapering to point, each bearing rows of downward-pointing sharp bristles, p5 dactylus shortest (Figs 2a, 3a).

**Figure 11.**
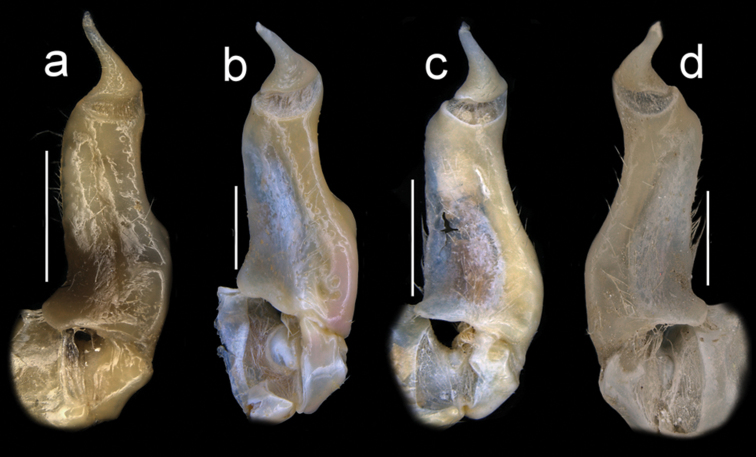
Four species of *Louisea* endemic to southwestern Cameroon, right G1 dorsal view (**a–c**), left G1 dorsal view (**d**). **a** Second largest adult male (CW 16.1 mm) of *L.edeaensis* from Lake Ossa wetland complex (ZMB Crust. 30335) **b** largest adult male (CW 16.2 mm) of *L.balssi* from Man’s Crater Lake Manengouba (ZMB Crust. 30319) (‘Mvogo Ndongo et al. 2018: fig. 5a’, www.mapress.com/j/zt) **c** adult male, holotype (CW 18.1 mm) of *L.yabassi* sp. nov. from Yabassi (ZMB Crust. 21575) **d** largest adult male, holotype (CW 20.0 mm) of *L.nkongsamba* sp. nov. from Mt. Nlonako (ZMB Crust. 31618). Scale bars: 2 mm (**a, c, d**), 1 mm (**b**).

**Figure 12.**
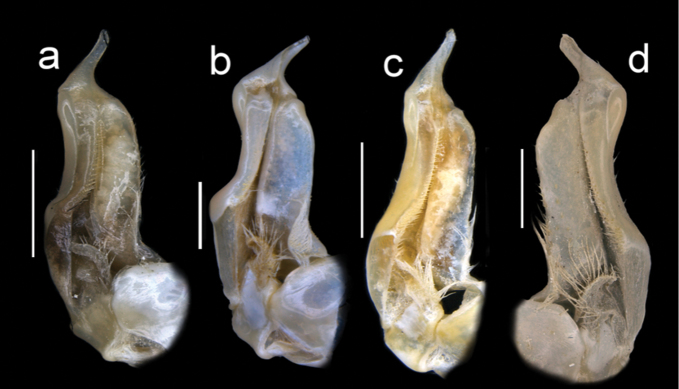
Four species of *Louisea* endemic to southwestern Cameroon, right G1 ventral view (**a–c**), left G1 ventral view (**d**). **a** Second largest adult male (CW 16.1 mm) of *L.edeaensis* from Lake Ossa wetland complex (ZMB Crust. 30335) **b** largest adult male (CW 16.2 mm) of *L.balssi* from Man’s Crater Lake Manengouba (ZMB Crust. 30319) (‘Mvogo Ndongo et al. 2018: fig. 5b’) **c** adult male, holotype (CW 18.1 mm) of *L.yabassi* from Yabassi (ZMB Crust. 21575) **d** largest adult male, holotype (CW 20.0 mm) of *L.nkongsamba* sp. nov. from Mt. Nlonako (ZMB Crust. 31618). Scale bars: 2 mm (**a, c, d**), 1 mm (**b**).

**Figure 13.**
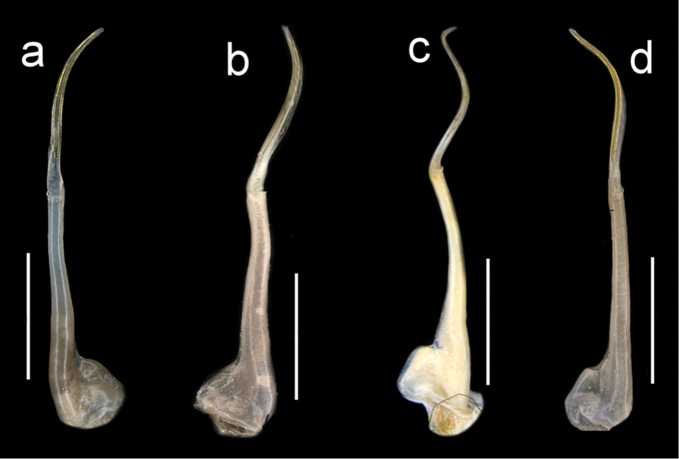
Four species of *Louisea* endemic to southwestern Cameroon, G2**a** second largest adult male (CW 16.1 mm) of *L.edeaensis* from Lake Ossa wetland complex (ZMB Crust. 30335) **b** largest adult male (CW 16.2 mm) of *L.balssi* from Man’s Crater Lake Manengouba (ZMB Crust. 30319) **c** adult male, holotype (CW 18.1 mm) of *L.yabassi* sp. nov. from Yabassi (ZMB Crust. 21575) **d** largest adult male, holotype (CW 20.0 mm) of *L.nkongsamba* sp. nov. from Mt. Nlonako (ZMB Crust. 31618). Scale bars: 2 mm (**a–d**).

Male pleon triangular, telson (a7) rounded at distal margin (Fig. 5a). G1TA short (TA/SS 0.3), directed outward at 45° angle to longitudinal axis of G1SS, proximally distinctly broad, abruptly narrow, slim and tube-like at distal two-thirds (Figs 11a, 12a). G1SS tapering slightly from broad basal margin to relatively wide distal margin (0.6 × SS basal margin); dorsal face with broad dorsal membrane (maximum width 0.1 ×SS length) at TA/SS junction (Fig. 11a); ventral face with raised triangular flap extending halfway across segment forming roof of chamber for G2, flap tapering diagonally from broad base to narrow point at SS/TA junction (Fig. 12a). G2TA long (TA/SS = 0.40), flagellum-like, almost as long as G2SS (Fig. 13a); G2SS wide at base, tapering sharply to long, thin process with raised rim at junction with TA (Fig. 13a). Mature between CW 14–23 mm.

###### Remarks.

The description and diagnosis of *L.edeaensis* by Cumberlidge (1994) was based on characters of specimens from Edea and Yabassi. Mvogo Ndongo et al. (2017a) updated these characters following the discovery of a large series of *L.edeaensis* from Bedimet Island in Lake Ossa in August 2015. These specimens included only one adult male that agreed well with the diagnostic characters of the holotype from Edea, and with other specimens from Yaounde and Yabassi. Nevertheless, the morphological variations raised by Cumberlidge (1994) that distinguished the specimens from Yabassi from those from Yaounde, Edea, and Lake Ossa still remained. The specimens of Lake Ossa examined in this study included five adult males whose morphological characters are consistent with those from Edea and Yaounde, but different from the specimens from Yabassi. This resulted in the present re-description of *L.edeaensis*. Differences between *L.edeaensis* and its congeners are given below under general remarks.

##### Louisea balssi

Taxon classificationAnimaliaDecapodaPotamonautidae

(Bott, 1959)

710D45B5-BAE1-50C4-B497-98CDDBE05016

Figs 2b , 3b , 4b , 5b , 6b , 7b , 8c, d , 9b , 10b , 11b , 12b , 13b , 14b , 15b

Globonautes balssi Bott 1959: 999, fig. 7; Cumberlidge 1987: 2210; 1994: 127, figs 2 a, b, 3 (j–l only), tables 1–2 (ZIM K 3506 only).
Globonautes macropus balssi Bott 1970: 25, pl. 1, figs 6–8.
Louisea balssi Cumberlidge 1999: 231, figs 53EE, 54–57, 62G, 68F, tables V, X–XIII, pl. 4 (not figs 46G, 48F, 49G, 52G, table IX); Ng et al. 2008: 169 (list); Mvogo Ndongo et al. 2018: 400.

###### Material examined.

CAMEROON. Holotype: juvenile male (CW 12.5 mm), Barombi Mbo [formerly Johann Albrechtshöhe (Government Station Johann Albrecht Mountain), Barombi Station] (4.666686N, 9.392042E), 323 m asl, 10 September 1909, coll. Carl Rathke (ZIM K3506). Paratypes: 3 adult females (CWs 22.0, 21.0, 21.0 mm) (ovigerous), subadult female (CW 13.5 mm), Barombi Mbo [formerly Johann Albrechtshöhe (Government Station Johann Albrecht Mountain), Barombi Station] (4.666686N, 9.392042E), 323 m asl, 10 September 1909, coll. Carl Rathke (SMF 5093, donated by ZIM K3506). Other material examined is given in Table 3.

**Table 3.** Morphometric analysis and collection data of specimens (*N* = 8) of *Louiseabalssi* from Cameroon (S.R, Man. Man’s Crater Lake; 5°01'56.9"N, 9°49'37.8"E; 1,958 m a.s.l.). All measurements are given in mm.

Specimens	CW	CL	CH	FW	CW/FW	CL/FW	CH/FW	FW/CL	Coll. Date	Museum
1 adult ♂	16.2	11.8	7.1	5.5	2.94	2.14	1.29	0.46	P.A.M.N 14.03.17	ZMB Crust. 30319
2 adult ♂	14.8	10.7	6.1	5.1	2.90	2.09	1.19	0.47	P.A.M.N 14.03.17	ZMB Crust.29628
3 adult ♂	14.3	10.5	6.0	5.0	2.86	2.10	1.20	0.47	P.A.M.N 14.03.17	LZUY 20 (IFAS-005)
4 adult ♂	13.3	9.7	5.6	4.6	2.89	2.11	1.21	0.47	P.A.M.N 14.03.17	LZUY 20 (IFAS-005)
5 adult ♀	14.8	10.9	6.4	4.7	3.14	2.31	1.36	0.43	P.A.M.N 14.03.17	ZMB Crust. 30319
6 subadult ♂	11.1	8.6	4.8	4.2	2.64	2.04	1.14	0.48	P.A.M.N 14.03.17	LZUY 20 (IFAS-005)
7 subadult ♂	12.7	9.2	5.7	4.3	2.95	2.13	1.32	0.46	P.A.M.N 14.03.17	LZUY 20 (IFAS-005)
8 subadult ♀	11.2	8.2	4.8	4.0	2.80	2.05	1.20	0.48	P.A.M.N 14.03.17	LZUY 20 (IFAS-005)
**Mean**	**13.6**	**10**	**5.8**	**4.7**	**2.89**	**2.12**	**1.23**	**0.47**	–	–

Key: P.A.M.N: Pierre A. Mvogo Ndongo

###### Diagnosis.

**Figure 14.**
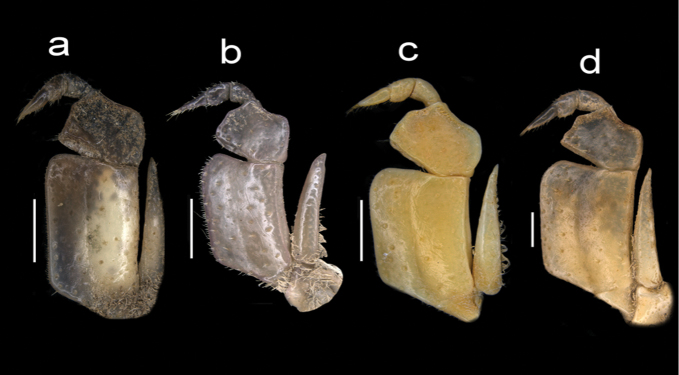
Four species of *Louisea* endemic to southwestern Cameroon, left third maxilliped. **a** Second largest adult male (CW 16.1 mm) of *L.edeaensis* from Lake Ossa wetland complex (ZMB Crust. 30335) **b** largest adult male (CW 16.2 mm) of *L.balssi* from Man’s Crater Lake Manengouba (ZMB Crust. 30319) (‘Mvogo Ndongo et al. 2018: fig. 4a’, www.mapress.com/j/zt) **c** adult male, holotype (CW 18.1 mm) of *L.yabassi* sp. nov. from Yabassi (ZMB Crust. 21575) **d** largest adult male, holotype (CW 20.0 mm) of *L.nkongsamba* sp. nov. from Mt. Nlonako (ZMB Crust. 31618). Scale bars: 2 mm (**a–d**).

**Figure 15.**
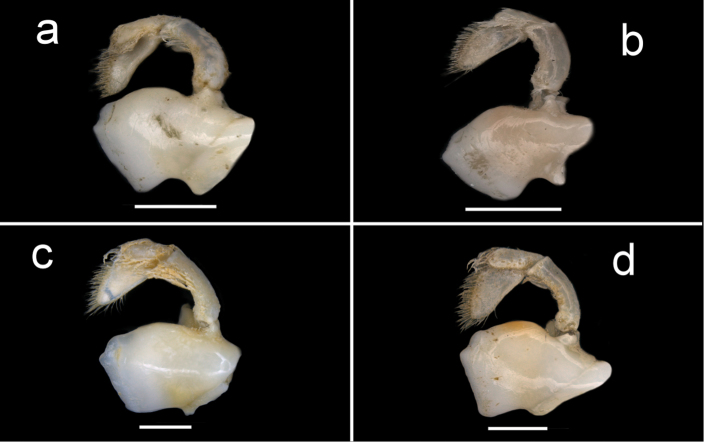
Four species of *Louisea* endemic to southwestern Cameroon, ventral view of right mandible. **a** Second largest adult male (CW 16.1 mm) of *L.edeaensis* from Lake Ossa wetland complex (ZMB Crust. 30335) **b** largest adult male (CW 16.2 mm) of *L.balssi* from Man’s Crater Lake Manengouba (ZMB Crust. 30319) **c** adult male, holotype (CW 18.1 mm) of *L.yabassi* sp. nov. from Yabassi (ZMB Crust. 21575) **d** largest adult male, holotype (CW 20.0 mm) of *L.nkongsamba* sp. nov. from Mt. Nlonako (ZMB Crust. 31618). Scale bars: 1 mm (**a, c**), 2 mm (**b, d**).

Amended from Cumberlidge (1994, 1999); Mvogo Ndongo et al. (2018). Carapace smooth, urogastric groove faint; postfrontal crest faint, complete, meeting anterolateral margin behind intermediate tooth (Fig. 3b); exorbital, intermediate teeth small, low, distinct (Figs 4b, 7b). Mandibular palp 2 segmented; terminal segment (TS) bilobed, with large distinct anterior lobe 0.5 × terminal segment length (Fig. 15b). Third maxilliped ischium with distinct vertical groove (Fig. 14b). Episternal sulci s4/e4, s5/e5, s6/e6 faint or missing, s7/e7 complete (Fig. 5b). Major cheliped dactylus relatively slender, highly arched enclosing oval interspace when closed, with two large teeth (one proximal, one medial) (Fig. 8b); propodus of major cheliped with two large proximal teeth, large medial tooth, small distal tooth (Fig. 8b); cheliped carpus inner margin with long, broad distal tooth, slim subequal proximal tooth (Fig. 10b); cheliped merus medial inferior margin with large jagged distal tooth followed by six distinct smaller teeth decreasing in size proximally (Fig. 9b). G1TA short (TA/SS 0.3), directed outwards at 45° angle to longitudinal axis of G1SS, with distinct longitudinal groove, proximally distinctly broad, abruptly narrow, slim and tube-like at distal two-thirds (Figs 11b, 12b). G1SS tapering slightly from wide basal margin to relatively wide distal margin (0.6 × SS basal margin); dorsal face with broad dorsal membrane (maximum width 0.1 × SS length) at TA/SS junction (Fig. 11b). G2TA long (TA/SS = 0.40), flagellum-like, almost as long as G2SS. Mature between CW 13–17 mm.

###### Redescription.

A re-description of *L.balssi* is given in Mvogo Ndongo et al. (2018). Additional character state descriptions are added here in the light of the new material to further distinguish between *L.balssi* and its congeners.

###### Remarks.

Specimens of *L.balssi* are known only from Kumba and Mt. Manengouba in southwestern Cameroon. The morphological features of *L.balssi* are mainly defined from the adult male specimens collected from Mt. Manengouba (Mvogo Ndongo et al. 2018). The only available specimens from Kumba are sub-adults whose gonopod, sternal, and cheliped characters are not fully developed, which makes them less useful for taxonomic studies (Cumberlidge 1999). Further systematic surveys at the type locality near Kumba are needed to resolve this taxonomic problem but current social issues in this part of Cameroon preclude such surveys. Distinctions between *L.balssi* and its congeners are given below under general remarks.

##### Louisea yabassi sp. nov.

Taxon classificationAnimaliaDecapodaPotamonautidae

55AE8B61-BEBF-5CFC-925B-97D5C7A7C0B5

http://zoobank.org/FA6DE8AD-B833-415C-95C7-47287F3C6158

Figs 2c , 3c , 4d , 5c , 6c , 7c , 8e, f , 9c , 10c , 11c , 12c , 13c , 14c , 15c

###### Material examined.

CAMEROON. Holotype: adult male (CW 18.11 mm, CL 12.78 mm, CH 8.30 mm, FW 6.29 mm; CW/FW 2.88, CL/FW 2.03, CH/FW 1.32, FW/CL 0.49, FW/CW 0.34), Yabassi, 10 September 1909, coll. Riggenbach (ZMB Crust. 21575). Paratype: subadult male (CW 13.82 mm, CL 10.61 mm, CH 6.25 mm, FW 5.01 mm; CW/FW 2.75, CL/FW 2.11, CH/FW 1.24, FW/CL 0.47, FW/CW 0.36), same data as holotype (ZMB Crust. 21575).

###### Diagnosis.

Carapace smooth, urogastric groove faint; postfrontal crest distinct, prominent, complete, meeting anterolateral margin behind intermediate tooth (Fig. 3c); exorbital, intermediate teeth large, triangular; epibranchial tooth undetectable (Figs 4c, 7c). Vertical sulcus on carapace branchiostegal wall curving backward to meet anterolateral margin at epibranchial tooth (Fig. 6c). Mandibular palp bi-segmented; terminal segment (TS) bilobed, with large distinct anterior lobe 0.6 × terminal segment length (Fig. 15c). Third maxilliped ischium with distinct vertical groove (Fig. 14c). Episternal sulci s4/e4, s5/e5, s6/e6 faint or missing, s7/e7 complete (Fig. 5c). Major cheliped dactylus highly arched enclosing oval interspace when closed, with five large teeth (one small distal, two large medial, two small proximal) (Fig. 8e); propodus of major cheliped with two large proximal teeth, large medial tooth, small distal tooth (Fig. 8e); cheliped carpus inner margin with long, broad distal tooth, relatively narrow, subequal proximal tooth (Fig. 10c). G1TA short (TA/SS 0.22), directed outwards at 45° angle to longitudinal axis of G1SS, with distinct longitudinal groove proximally distinctly broad, abruptly narrow, slim and tube-like at distal two-thirds (Figs 11c, 12c). G1SS tapering slightly from broad basal margin to relatively wide distal margin (0.5 × SS basal margin); dorsal face with broad dorsal membrane (maximum width 0.1 × SS length) at TA/SS junction (Fig. 11c). G2TA long (TA/SS 0.44), flagellum-like, almost as long as G2SS (Fig. 13c). Mature at CW 19 mm.

###### Description.

Carapace ovoid, flat (CH/FW 1.28, *N* = 2), wide (CW/FW 2.8), smooth, urogastric groove distinct; front wide (FW/CW 0.35, *N* = 2), deflexed, anterior margin straight; postfrontal crest distinct, prominent, completely crossing carapace, meeting anterolateral margin of carapace behind intermediate tooth (Fig. 4d); exorbital, intermediate teeth large, triangular; epibranchial tooth undetectable (Figs 4c, 7c). Carapace branchiostegal sidewall with vertical, longitudinal sutures dividing it into three regions (suborbital, subhepatic, pterygostomial) (Fig. 6c); longitudinal suture beginning at respiratory opening, curving backward across sidewall dividing suborbital- and subhepatic regions from pterygostomial region (Fig. 6c); vertical sulcus on carapace branchiostegal wall curving backward to meet anterolateral margin at epibranchial tooth (Fig. 6c), dividing suborbital from subhepatic regions (Fig. 6c).

Mandibular palp bi-segmented; terminal segment (TS) bilobed, with large distinct anterior lobe 0.6 × terminal segment length (Fig. 15c). Third maxilliped exopod completely lacking flagellum; ischium with distinct vertical groove (Fig. 14c). Sternal sulcus s2/s3 prominent, completely crossing sternum; s3/s4 incomplete, reduced to 2 short lateral notches (Fig. 5c). Episternal sulci s4/e4, s5/e5, s6/e6 faint or missing, s7/e7 complete (Fig. 5c).

Male chelipeds unequal, right chelipeds larger than left cheliped; fingers slim, elongated. Right (major) cheliped dactylus relatively stout, straight, with five large teeth (one small distal, two large medial, two small proximal); fixed finger (pollex) with five large teeth (one distal, four proximal) (Fig. 8e); dactylus and propodus of left cheliped straight, with small teeth on occluding margin (Fig. 8f); cheliped carpus inner margin with two pointed teeth, distal tooth large with pointed tip, proximal tooth smaller (Fig. 10c); medial inferior margin of cheliped merus with large jagged distal tooth followed by numerous distinct smaller teeth (Fig. 9c).

Male pleon triangular, sides not indented with small setae; telson (a7) rounded at distal margin (Fig. 5c). G1TA short (TA/SS 0.22), directed outwards at 45° angle to longitudinal axis of G1SS, with distinct longitudinal groove on ventral face, proximally distinctly broad, abruptly narrow, slim and tube-like at distal two-thirds (Figs 11c, 12c). G1SS tapering slightly from broad basal margin to relatively wide distal margin (0.5 × SS basal margin); ventral face of with raised triangular flap extending halfway across segment forming roof of chamber for G2, flap tapering diagonally from broad base to narrow point at SS/TA junction (Fig. 12c); dorsal face with broad dorsal membrane (maximum width 0.1 × SS length) at TA/SS junction (Fig. 11c). G2TA long (TA/SS 0.44), flagellum-like, almost as long as G2SS (Fig. 13c). Mature at CW 19 mm.

###### Color in life.

Unknown.

###### Type locality.

Yabassi, littoral region of Cameroon.

###### Etymology.

The new species is named for Yabassi in south-western Cameroon, where it was collected in 1909. The species epithet is a noun in apposition.

###### Habitat.

*Louiseayabassi* sp. nov. is known only from Yabassi, a humid area of the coastal rain forest of southwestern Cameroon. It is possible that this species is also present in the Ebo forest near Yabassi, which is one of the largest remaining tracts of lowland and submontane rainforest in the area.

###### Remarks.

Characters of the carapace and chelipeds of adult male specimens from Yabassi assigned to *L.edeaensis* by Cumberlidge (1994) and by Mvogo Ndongo et al. (2017a) proved to be inconsistent with the holotype from Edea. Re-examination of these specimens supported the hypothesis that specimens from Edea and those from Yabassi belong to two different species: *L.edeaensis* (Edea, Yaounde, and Lake Ossa) and *L.yabassi* sp. nov. (Yabassi). The differences between *L.yabassi* sp. nov. and its congeners are discussed below under general remarks.

##### Louisea nkongsamba sp. nov.

Taxon classificationAnimaliaDecapodaPotamonautidae

393AB7F6-A16A-5514-9098-E1A1C9709C6C

http://zoobank.org/141A1FD3-DF3B-4E84-9296-5AA5A26A3B68

Figs 2d , 3d , 4a , 5d , 6d , 7d , 8g, h , 9d , 10d , 11d , 12d , 13d , 14d , 15d

###### Material examined.

CAMEROON. Holotype: adult male (CW 20 mm, CL 14.85 mm, CH 8.4 mm, FW 6.6 mm), Littoral Region, Mount Nlonako Ecological Reserve (locality 1) (4.91046N, 9.976332E), 1,237 m asl, 23 May 2018, coll. P.A. Mvogo Ndongo (ZMB Crust. 31618). Paratype: adult male (CW 18.38 mm, CL 13.32 mm, CH 8.13 mm, FW 6.34 mm), Littoral Region, Mount Nlonako Ecological Reserve (locality 2) (4.91343N, 9.98500E), 1,176 m asl, 23 May 2018, coll. P.A. Mvogo Ndongo (ZMB Crust. 31620). Other material examined is listed in Table 4.

**Table 4.** Morphometric analysis and collection data of specimens (*N* = 27) of *Louiseankongsamba* sp. nov. from Cameroon. All measurements are given in mm.

**Specimens**	**CW**	**CL**	**CH**	**FW**	**CW/FW**	**CL/FW**	**CH/FW**	**FW/CL**	**Coll. Date**	**Museum**
1 ad ♂^1^	20	14.85	8.4	6.6	3.03	2.25	1.27	0.44	P.A.M.N 23.05.18	ZMB Crust. 31618
2 ad ♂^2^	18.38	13.32	8.13	6.34	2.89	2.1	1.28	0.47	P.A.M.N 25.05.18	ZMB Crust. 31620
3 ad ♂^1^	18.36	13.30	8.12	6.17	2.97	2.15	1.31	0.46	P.A.M.N 23.05.18	ZMB Crust. 31618
4 ad ♂^3^	17.01	12.30	7.53	5.56	3.05	2.21	1.35	0.45	P.A.M.N 26.05.18	ZMB Crust. 31619
5 ad ♂^3^	17.27	12.80	7.55	5.50	3.14	2.32	1.36	0.42	P.A.M.N 26.05.18	ZMB Crust. 31619
6 ad ♂^4^	18.40	13.52	8.56	5.90	3.11	2.29	1.45	0.32	P.A.M.N 27.05.18	ZMB Crust. 31621
7 ad ♂^4^	17.61	13.15	7.80	6.08	2.89	2.16	1.28	0.34	P.A.M.N 27.05.18	ZMB Crust. 31621
8 ad ♂^4^	17.37	12.89	7.73	5.60	3.10	2.30	1.38	0.43	P.A.M.N 27.05.18	ZMB Crust. 31621
9 ad ♂^1^	18.05	12.84	8	6.16	2.93	2.08	1.29	0.47	P.A.M.N 25.05.18	ZMB Crust. 31618
10 ad ♂^1^	16.75	12.50	7.71	5.77	2.90	2.16	1.33	0.46	P.A.M.N 23.05.18	ZMB Crust. 31618
11 ad ♂^1^	16.01	11.68	7.04	5.31	3.01	2.19	1.32	0.45	P.A.M.N 23.05.18	ZMB Crust. 31618
12 ad ♂^1^	16.90	12.20	7.81	5.32	3.17	2.29	1.46	0.43	P.A.M.N 23.05.18	ZMB Crust. 31618
13 ad ♂^1^	15.24	11.47	6.41	5.42	2.81	2.11	1.18	0.47	P.A.M.N 23.05.18	ZMB Crust. 31618
14 ad ♂^2^	15.55	11.60	6.93	5.40	2.87	2.14	1.28	0.46	P.A.M.N 25.05.18	ZMB Crust. 31620
15 ad ♂^2^	16.50	12.19	7.47	5.60	2.94	2.17	1.33	0.45	P.A.M.N 25.05.18	ZMB Crust. 31620
16 ad ♀^2^	19.72	14.76	9.12	7.03	2.80	2.1	1.29	0.47	P.A.M.N 25.05.18	IFAS-005
17 ad ♀^2^	17.10	13.03	8.28	5.96	2.86	2.18	1.38	0.45	P.A.M.N 25.05.18	IFAS-005
18 ad ♀^1^	16.15	11.66	7.10	5.62	2.87	2.07	1.26	0.48	P.A.M.N 23.05.18	IFAS-005
19 ad ♀^2^	15.04	11.10	6.86	5	3	2.22	1.37	0.45	P.A.M.N 25.05.18	IFAS-005
20 ad ♀^2^	14.73	10.80	6.25	4.80	3.06	2.25	1.30	0.44	P.A.M.N 25.05.18	IFAS-005
21 ad ♀^2^	14.56	10.9	6.08	5.15	2.82	2.11	1.18	0.47	P.A.M.N 25.05.18	IFAS-005
22 sd ♂^2^	13.49	10.33	6.20	4.45	3.03	2.32	1.39	0.43	P.A.M.N 25.05.18	IFAS-006
23 sd ♂^2^	13.76	10.54	5.85	4.35	3.16	2.42	1.34	0.41	P.A.M.N 25.05.18	IFAS-006
24 sd ♂^2^	13.33	10.30	6.10	4.25	3.13	2.42	1.43	0.41	P.A.M.N 25.05.18	IFAS-006
25 sd ♂^2^	12.87	10.08	5.86	4.17	3.08	2.41	1.40	0.41	P.A.M.N 25.05.18	IFAS-006
26 sd ♂^2^	13.14	10.09	5.91	4.60	2.85	2.19	1.28	0.45	P.A.M.N 25.05.18	IFAS-006
27 sd ♂^2^	12.91	9.94	5.64	4.70	2.74	2.11	1.20	0.47	P.A.M.N 25.05.18	IFAS-006
**Mean**	**16.15**	**12.00**	**7.20**	**5.46**	**2.95**	**2.19**	**1.32**	**0.45**	–	–

Key: P.A.M.N: Pierre A. Mvogo Ndongo; ad: adult; sd: subadult. Location: ^1^ Nlonako site 1; 4°54'44.8"N, 9°58'50.2"E; 1211 m a.s.l.; ^2^ Nlonako site 2; 4.91343°N, 9.98500°E; 1176 m a.s.l.; ^3^ Nlonako site 3; 4°53'30.5"N, 9°59'12.1"E; 938 m a.s.l.; ^4^ Nlonako site 4; 4°54'56.4"N, 9°59'41.8"E ASL: 1392 m a.s.l.

###### Diagnosis.

Carapace smooth, urogastric groove faint; postfrontal crest faint, complete, meeting anterolateral margin behind intermediate tooth (Fig. 7d); exorbital, intermediate teeth large, triangular; epibranchial tooth undetectable (Figs 4d, 7d). Vertical sulcus on carapace branchiostegal wall meeting anterolateral margin at intermediate tooth (Fig. 6d). Mandibular palp bi-segmented; terminal segment (TS) bilobed, with large distinct anterior lobe 0.8 × terminal segment length (Fig. 15d). Third maxilliped exopod completely lacking flagellum; ischium with distinct vertical groove (Fig. 14d). Episternal sulci s4/e4, s5/e5, s6/e6, s7/e7 complete (Fig. 5d). Major cheliped dactylus relatively stout, straight, not arched, enclosing long thin interspace when closed, with small distal tooth (Fig. 8g); propodus of major cheliped with three large teeth (proximal, medial, distal) (Fig. 8g); cheliped carpus inner margin with large, broad pointed distal tooth, robust subequal proximal tooth (Fig. 10d); cheliped merus medial inferior margin with large jagged distal tooth followed by several distinct smaller teeth (Fig. 9d). G1TA short (TA/SS 0.22), directed outwards at 45° angle to longitudinal axis of G1SS, with distinct longitudinal groove on ventral face, proximally distinctly broad, abruptly narrow, slim and tube-like at distal two-thirds (Figs 11d, 12d). G1SS tapering slightly from broad basal margin to relatively wide distal margin (0.5 × SS basal margin); dorsal face with broad dorsal membrane (maximum width 0.1 × SS length) at TA/SS junction (Fig. 11d). G2TA long (TA/SS 0.44), flagellum-like, almost as long as G2SS (Fig. 13d). Mature at CW 20 mm.

###### Description.

Carapace ovoid, moderately high (CH/FW 1.32, *N* = 27), wide (CW/FW 2.95, *N* = 27), texture smooth, urogastric groove distinct. Front wide (FW/CW 0.34, *N* = 27), deflexed, anterior margin straight (Figs 2d, 3d, 4a, 7d). Postfrontal crest faint but complete, ends meeting anterolateral margins at epibranchial teeth (Fig. 7d); mid-groove faint, shallow; epigastric crests poorly defined (Fig. 7d). Exorbital, intermediate teeth large, triangular, epibranchial tooth small but detectable (Figs 4a, 7d). Anterolateral margin of carapace lined by small granules (Figs 4a, 7d); posterolateral margin curving inward, continuous with anterolateral margin (Fig. 7d); posterior carapace margin about 2/3 CW. Carapace branchiostegal wall with longitudinal, vertical sutures dividing sidewall into three parts (Fig. 6d). Longitudinal sulcus beginning at respiratory opening, curving backward across sidewall, dividing suborbital- and subhepatic regions from pterygostomial region (Fig. 6d); vertical sulcus on sidewall marked by row of granules, meeting anterolateral margin at intermediate tooth (Fig. 6d), dividing suborbital- from subhepatic regions (Figs 6d, 7d). Sternal sulcus s2/s3, deep, ends not meeting side margins of sternum (Fig. 5d); s3 with distinct central depression; s3/s4 reduced to two short lateral notches (Fig. 5d). Episternal sulci s4/e4, s5/e5, s6/e6, s7/e7 complete (Fig. 5d). Mandibular palp bi-segmented; terminal segment (TS) bilobed, with large distinct anterior lobe 0.8 × terminal segment length (Fig. 15d). Third maxilliped (Fig. 6d) filling entire buccal cavern, except for transversely oval efferent respiratory openings in superior lateral corners; exopod completely lacking flagellum; ischium with distinct vertical groove (Fig. 14d).

Major cheliped dactylus relatively stout, straight, not arched enclosing long, thin interspace when closed, with small proximal tooth (Fig. 8g); propodus of major cheliped with three large teeth (proximal, medial, distal) (Fig. 8g); cheliped carpus inner margin with large, broad, pointed distal tooth, robust, subequal proximal tooth (Fig. 10d). Walking legs (p2–p5 slender, p4 longest, p5 shortest; dactyli (p2–p5) tapering to point, each bearing rows of downward-pointing sharp bristles, p5 dactylus shortest (Figs 2d, 3d).

Male pleon triangular, telson (a7) with rounded distal margin (Fig. 5d). G1TA short (TA/SS 0.22), directed outwards at 45° angle to longitudinal axis of G1SS, with distinct longitudinal groove on ventral face, proximally distinctly broad, abruptly narrow, slim and tube-like at distal two-thirds (Figs 11d, 12d). G1SS tapering slightly from broad basal margin to relatively wide distal margin (0.5 × SS basal margin); dorsal face with broad dorsal membrane (maximum width 0.1 × SS length) at TA/SS junction (Fig. 11d). G2TA long (TA/SS 0.44), flagellum-like, almost as long as G2SS (Fig. 13d).

###### Color in life.

Specimens of *L.nkongsamba* sp. nov. have a dark brown or green carapace and walking legs.

###### Type locality.

Nlonako Wildlife Reserve, Nkongsamba, littoral region of Cameroon.

###### Etymology.

The new species is named for Nkongsamba, the closest town to the type locality. The species epithet is a noun in apposition.

###### Habitat.

*L.nkongsamba* sp. nov. is known only from Nlonako Wildlife Reserve, one of the threatened tropical rainforest habitats in the littoral region of Cameroon.

###### Remarks.

*L.nkongsamba* sp. nov. possesses numerous characters that link it to *L.edeaensis*, *L.balssi*, and *L.yabassi* sp. nov. Differences between these species are discussed below under general remarks.

**Figure 16.**
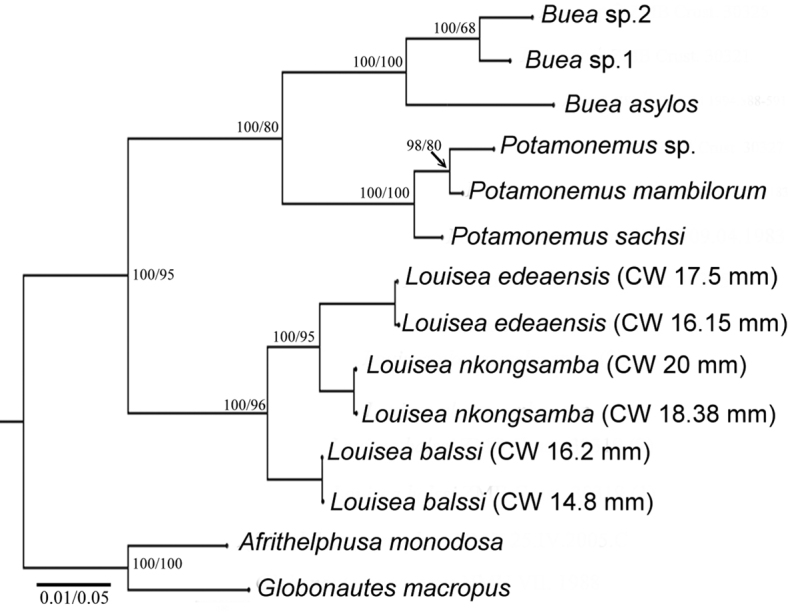
ML tree topology for the freshwater crab taxa from Cameroon included in this study derived from mtDNA sequences corresponding to three loci (partial 16S rRNA, COI and 12S rRNA genes). BI and ML statistical values (%) on the nodes indicate posterior probabilities and bootstrap support, respectively.

#### General remarks

The generic characters of *Louisea* proposed by Cumberlidge (1994, 1999) were based on *L.edeaensis* and a subadult specimen of *L.balssi*. These characters are therefore reassessed here in the light of recently-rediscovered populations of *L.edeaensis* and *L.balssi* that included adult males of both species (Mvogo Ndongo et al. 2017a, 2018), and the two new species from Yabassi and Mt. Nlonako described here. All these four species are assigned to *Louisea* because they share the emended diagnostic characters for the genus presented here, and three of them at least form a monophyletic group (Fig. 16). The main characters that differentiate the four species are based on the cheliped dactylus; the inner margin teeth on the cheliped carpus; the intermediate tooth between exorbital and epibranchial teeth; the postfrontal crest; sternal segment S3; sternal sulcus S2/S3; the anterior lobe on the TS of the mandibular palp; and the G1TA length. Some of these characters are shared by two of the four species. The differences between the four species of *Louisea* are given in Table 5.

**Table 5.** Differences between the four species of the genus *Louisea*.

Characters	*L.nkongsamba* sp. nov.	*L.yabassi* sp. nov.	*L.balssi*	*L.edeaensis*
Major cheliped dactylus shape	Stout, straight (Fig. 8h)	Stout, straight (Fig. 8e)	Slender, highly arched (Fig. 8c)	Stout, straight (Fig. 8a)
Major cheliped propodus dentition	3 large teeth (Fig. 8h)	5 large teeth (Fig. 8e)	2 large proximal teeth (Fig. 8c)	4 large teeth (Fig. 8a)
Cheliped carpus inner margin teeth	Distal larger than proximal (Fig. 10d)	Both large and pointed (Fig. 10c)	Both large and pointed (Fig. 10b)	Distal larger than proximal (Fig. 10a)
Intermediate tooth between exorbital and epibranchial teeth	Large and triangular (Figs 4a, 7d)	Large and triangular (Figs 4d, 7c)	Faint, barely detectable (Figs 4b, 7b)	Small but distinct (Figs 4c, 7a)
Postfrontal crest	Faint (Figs 4a, 7d)	Prominent, clearly defined (Figs 4d, 7c)	Faint (Figs 4b, 7b)	Faint (Figs 4c, 7a)
Sternal sulcus s2/s3	Deep, ends not meeting side margins of sternum (Figs 5d, 6d)	Deep, ends meeting side margins of sternum (Figs 5c, 6c)	Deep, ends meeting side margins of sternum (Figs 5b, 6b)	Faint and shallow, ends meeting side margins of sternum (Figs 5a, 6a)
Sternal segment s3	With distinct central depression (Figs 5d, 6d)	No depression (Figs 5c, 6c)	No depression (Figs 5b, 6b)	No depression (Figs 5a, 6a)
Size of anterior lobe on terminal segment (TS) of mandibular palp	Large (0.8× TS length) (Fig. 15d)	Medium (0.6× TS length) (Fig. 15c)	Small (0.5× TS length) (Fig. 15b)	Medium (0.6× TS length) (Fig. 15a)
G1TA length	Short (0.22× G1 length) (Fig. 11d)	Medium (0.27× G1 length) (Fig. 11c)	Medium (0.28× G1 length) (Fig. 11b)	Medium (0.29× G1 length) (Fig. 11a)

## Discussion

The highlands of southwestern Cameroon are part of the continental segment of the Cameroon Volcanic Line (CVL) that includes Mount Cameroon, Mt. Manengouba, Mt. Nlonako, Mt. Lefo, Mt. Oku, and the Ngaoundere Plateau (Burke 2001; Zimkus 2009). The CVL crosses a significant portion of the West African forest biodiversity hotspot, which is remarkably species rich and has a high rate of endemism (Stuart 1986; Lawson 1993; Bowden and Andrews 1994; Stattersfield et al. 1998; Myers et al. 2000; Lovett and Taplin 2004; Herrmann et al. 2005). Although the freshwater crab fauna of southwestern Cameroon is also species-rich and has a high number of endemic species (Cumberlidge et al. 2019), most parts of this part of the country have remained largely unsurveyed for freshwater crabs until recently (Mvogo Ndongo et al. 2017a, 2017b, 2017c, 2018). Recent biotic surveys of the freshwater crab fauna in southwestern Cameroon coupled with the re-examination of museum specimens have led to the discovery or rediscovery of a number of taxa, raising the number of species from Cameroon to 17, and the number of genera to five (Cumberlidge et al. 2019). However, the two rediscovered species *Louiseaedeaensis* and *L.balssi* and the two new species of *Louisea* described here are all from new localities, because attempts to find additional specimens of *Louisea* were not successful at Yabassi, Edea, Yaounde and Kumba, localities where they were collected between 1900 and 1910. It is possible that the populations of *Louisea* in these four locations have been extirpated because they were last found in 1910, and there has been considerable human population expansion and urban development in these areas since then. This is especially unfortunate because the specimens from Yabassi have a problematic taxonomic history, but the larger series of specimens of all species in this genus now available enables us to recognise the specimens from Yabassi as a new species, *L.yabassi* sp. nov.

Our phylogenetic analyses (Fig. 16) based on 1801 base pairs of three mitochondrial genes (combined COI, 16S RNA, 12S RNA) found strong BI and ML support for the continued recognition of the genus *Louisea* with a well-supported clade that includes *L.edeaensis*, *L.balssi*, and *L.nkongsamba* sp. nov. We were not able to extract DNA from the available specimens of *L.yabassi* sp. nov., and this species was therefore not included in the tree. It has been suggested that the montane regions on the Cameroon Volcanic Line act as centres of speciation, as has been reported by Zimkus (2009) for puddle frogs from Mt. Oku. Our data indicate that the highland dwelling species *L.balssi* is the sister group to the two other species of *Louisea* found at lower altitudes, which indicates that speciation may have happened along an altitudinal gradient. However, further phylogenetic analyses are needed to establish whether this can lend support to the hypothesis that CVL is a montane centre of speciation. It is interesting to note that our phylogenetic tree also recognizes for the first time two distinct lineages within the genus *Buea* and one lineage within *Potamonemus* (Mvogo Ndongo et al. in prep). *Buea* and *Potamonemus* are sister genera and are apparently derived from the well-established diversified lineages of *Louisea*, but a phylogenetic work that includes all known genera assigned to the Potamonautinae is needed to test this hypothesis.

### Revised key to the species of the genus *Louisea* Cumberlidge, 1994

**Table:** 

1	Postfrontal crest prominent, clearly defined (Figs 4d, 7c)	***L.yabassi* sp. nov.**
–	Postfrontal crest faint (Figs 4a–c, 7a, b, d)	**2**
2	Dactylus of male major cheliped relative slender, highly arched (Fig. 8c)	***L.balssi***
–	Dactylus of male major cheliped relative stout, straight (Fig. 8a, h)	**3**
3	Sternal segment s3 lacking central depression, sternal sulcus s2/s3 relatively shallow, ends meeting side margins of sternum (Figs 5a, 6a); anterior lobe on terminal segment of mandibular palp relatively small, 0.6× TS length (Fig. 15a); G1 terminal article relatively long (TA/SS 0.3) (Fig. 11a)	***L.edeaensis***
–	Sternal segment s3 with distinct central depression, sternal sulcus s2/s3 deep, ends not meeting side margins of sternum (Figs 5d, 6d); anterior lobe on terminal segment of mandibular palp relatively large, 0.8× TS length (Fig. 15d); G1 terminal article relatively short (TA/SS 0.2) (Fig. 11d)	***L.nkongsamba* sp. nov.**

## Supplementary Material

XML Treatment for Louisea

XML Treatment for Louisea edeaensis

XML Treatment for Louisea balssi

XML Treatment for Louisea yabassi

XML Treatment for Louisea nkongsamba
